# The Art of Professional Teaching

**Published:** 1896-05

**Authors:** 


### Editorial.

### THE ART OF PROFESSIONAL TEACHING.

The training of the young and the education of the more mature has been a subject of thought for the best minds, both in literary, scientific, and professional circles. Theories have been framed and lectureships established to meet a growing demand for thoroughness in this important matter. In Europe, and especially in Germany where the science and art of education is best understood, this is held to be of so much consequence to the good or ill of the developing mind that pedagogy has become a necessary part of the educational system. It has not been carried to the same extent in this country, and the unfortunate idea still prevails that any one is capable of teaching provided he or she possesses the necessary knowledge in any given line of work. Upon this mistaken conception the educational process, while good and in some respects superior, has proceeded with varied results until the United States has practically no system as compared with other countries, and no proper organization of methods in training.

This weakness in our school methods is deplored by many who have been able to make some study of more thorough systems, but there seems no immediate prospect of anything better being evolved from the practices of the past and present.

So thoroughly has the idea, that any one can teach, become part of the American thought that any assertion that something else than mere knowledge is required is regarded as largely the production of senile crudities and not a part of the active progressive thought of the age.

While the foregoing remarks apply to the general work of the teacher, it must not be understood that they do not have force in all departments of training. The defective ideas alluded to very naturally extend into every channel of development; in fact, it is not asked in professional circles how does he teach, but what does he know. When a man becomes an applicant for a teacher's position, in Germany, he must prove his ability in that direction. It is not regarded as sufficient that he knows a thing, he must show that he is capable of imparting it.

If this were adopted in the professional schools in this country there would certainly be much gained, and a general advance would be experienced along all lines of professional work.

The mistake is being continually made that men are taken from the ranks and given positions as educators without a particle of experience to guide them. The result is generally a mortification to the pride of the selected aspirant, and a positive injury to the unfortunate students. Occasionally one may be found so gifted by nature as to be able to overcome the absence of systematic training, just as we find, very occasionally, an actor may make a name on the stage who has never worked his way step by step through subordinate positions. As actors are not created solely by this most lengthened process of training, but must have the gift by nature, so teachers cannot be developed through the same method, though they may be improved and rendered of some value by constant practice.

We have been led into this train of thought by the great multiplication of technical schools especially in our profession. The number has grown so rapidly that almost every State in the Union has one or more, and to these constant additions are being made. This rapid multiplication means a demand for professional teachers, and these must perforce be taken from the ranks of practitioners.

Hence colleges are chartered and opened by men whose entire knowledge is based on two or three years of undergraduate experience to which has been added a certain period of practice. The power to impart has never been learned; indeed, in many cases the newly-fledged professor finds difficulty in maintaining his position without loss of self-respect, or, what is of more importance, the loss of confidence of the student.

This elevation of the individual, without previous preparatory work, means always stilted methods, talking over the heads of his auditors, an incapacity to meet the simplest mind among his listeners. The power to work in sympathetic unison with the learner is primarily the important thing required of a teacher, and without this ability the newly-titled professor will exhibit wisdom by retiring, and returning to his former practical vocation.

It is not unusual to find the suddenly elevated man making his first great mistake in the use of ponderous language and technical terms without limit. No greater error can be made than this. The simplest form of expression is always the best, either in writing or speaking, and this is imperatively necessary in teaching.

This fault is not confined to the lecture-room, but has been translated from this locality to the general work of the essayist. Papers are made tedious and very unclear by the overloading with technicalities and coined words, the writers evidently imagining that profundity is in proportion to redundancy of expression and multiplication of sylables.

That which is especially needed, not only in our profession but in all others, is the thorough training of those who aspire to be teachers, and this, in dental work, must begin with the demonstrator. In this position he should be able to show the qualities he possesses and whether they will fit him for enlarged work and heavier responsibilities. The time is past for men to be given the highest positions who are ignorant of the first principles required in the formation of professional character or practical excellence. Whether dental colleges of the future will be regulated according to the system adopted by those well versed in the best traditions of pedagogy, it is certain, if good results are to be attained, those who aspire to become instructors must serve before they can lead, and wait humbly for that development which only comes, if it comes at all, through an intelligent use of experience.

